# Analysis of the Molecular Mechanism of Acute Coronary Syndrome Based on circRNA-miRNA Network Regulation

**DOI:** 10.1155/2020/1584052

**Published:** 2020-04-29

**Authors:** Fei Lin, YaMing Yang, Quan Guo, Mingzhang Xie, Siyu Sun, Xiulong Wang, Dongxu Li, Guhao Zhang, Meng Li, Jie Wang, Guoan Zhao

**Affiliations:** ^1^The First Hospital of Xinxiang Medical University, Xinxiang, Henan 453100, China; ^2^Heart Center of Xinxiang Medical University, Xinxiang, China; ^3^Henan Engineering Research Center for Clinical Data and Biobank of Cardiovascular Diseases, Zhengzhou, Henan, China; ^4^Guang'anmen Hospital, China Academy of Chinese Medical Sciences, Beijing 100053, China

## Abstract

**Background:**

With the development of biological technology, biomarkers for the prevention and diagnosis of acute coronary syndrome (ACS) have become increasingly evident. However, the study of novel circular RNAs (circRNAs) in ACS is still in progress. This study aimed to investigate whether the regulation of circRNA-miRNA networks is involved in ACS pathogenesis.

**Methods:**

We used microarray analysis to detect significantly expressed circRNAs and miRNAs in the peripheral blood of patients in the control group (CG) and ACS groups, including an unstable angina pectoris (UAP) group and an acute myocardial infarction (AMI) group. A circRNA-miRNA interaction network analysis was carried out with open-source bioinformatics. The gene ontology (GO), pathway, and disease enrichment analyses for differentially expressed circRNAs were further analysed with hierarchical clustering.

**Results:**

A total of 266 circRNAs (121 upregulated and 145 downregulated, *P* < 0.05, fold change FC ≥2) and 3 miRNAs (1 upregulated and 2 downregulated, *P* < 0.05, FC ≥ 1.2) were differentially expressed in the ACS groups compared with those in the CG. In addition, among these expressed circRNAs and miRNAs, a single circRNA could bind to more than 1–100 miRNAs, and vice versa. Next, an AMI-UAP network, an AMI-CG network, a UAP-CG network, and an AMI-CG-UAP network were constructed. The top 30 enriched GO terms among the three groups were emphasized as differentially expressed. Disease enrichment analysis showed that these differentially expressed circRNAs are involved in the pathogenesis of cardiovascular diseases. KEGG pathway analysis was performed to identify pathways associated with circRNAs targeting mRNAs.

**Conclusion:**

CircRNAs are closely related to the pathological process of ACS via a mechanism that may be related to the up- or down-regulation of circRNAs and miRNAs and circRNA-miRNA coexpression. The metabolic pathways, signalling pathways, and diseases affected by these circRNAs can be predicted by enrichment analysis.

## 1. Introduction

Circular RNAs (circRNAs), which contain a covalently closed continuous loop, are an abundant class of endogenous RNAs that are formed during the maturation of precursor mRNA. CircRNAs are widely expressed in eukaryotes, are evolutionarily conserved, and can be specific to certain cell types or developmental stages. In addition, circRNAs have been found in the nucleus and mitochondria. Unlike linear RNA, circRNAs have no 5′ cap or 3′ tail structure and is not easily degraded by the exonuclease RNase R, which is stable in cells [1–3]. The formation mechanism of circRNAs also determines its diverse and complex biological functions [4–7]. Among them, its characteristics of transcription, translation, protein interaction, and signal transduction regulation have been confirmed by a growing number of studies [3, 8]. In particular, circRNAs can specifically change the biological behaviour of cells in tissues and in diseases [3]. Moreover, many studies have shown that circRNAs are widely and specifically expressed in tumours, ageing, diabetes, cardiovascular and cerebrovascular diseases, and skin diseases [1, 7, 9–11]. These results offer a new perspective on biomolecular science.

Acute coronary syndrome (ACS) can lead to a series of acute cardiovascular events, such as arrhythmia, heart failure, and even sudden death. Its main pathogenesis is closely related to plaque rupture, vasospasm, platelet aggregation, and thrombosis. However, the mechanism remains unclear. With the rapid development of next-generation gene sequencing technology, an increasing number of reports have indicated that noncoding RNAs (ncRNAs), such as circRNAs, microRNAs (miRNAs), and long noncoding RNAs (lncRNAs), have a significant influence on cardiovascular diseases [12]. CircRNAs, as regulators of gene expression, may be an important genetic mechanism underlying the pathogenesis of multifactorial complex diseases [10, 13–16]. Thus, elucidating the process by which miRNAs regulate the gene expression and the specificity of the regulated targets is highly important for probing the mechanism underlying ACS [17]. However, few studies have investigated whether circRNAs and miRNAs are involved in the occurrence and development of ACS. The results of our previous work have indicated that circRNAs are significantly expressed in the blood of patients with coronary heart disease (CHD) [18].Thus, in this work, we screened the characteristic circRNA and miRNA expression profiles of ACS using a microarray gene chip and predicted the possible circRNA-miRNA interaction. We aimed to provide critical information for investigations into the complex regulatory mechanisms of ACS.

## 2. Materials and Methods

### 2.1. Study Subjects

We included inpatients diagnosed with ACS (I24.901), including those diagnosed with acute myocardial infarction (AMI) (I21) and unstable angina pectoris (UAP) (I20.001) according to the criteria of the International Classification of Diseases–10th edition, who were treated at the Department of Cardiology of the First Affiliated Hospital of Xinxiang Medical College between November 2016 and February 2017. The diagnostic criteria for AMI and UAP were based on the globally harmonized definition of AMI and on the 2012 American College of Cardiology Foundation (ACCF)/American Heart Association (AHA) Focused Update of the Guidelines for the Management of Patients with Unstable Angina/Non-ST-elevation Myocardial Infarction [19–21]. All participants underwent coronary angiography (CAG), and Gensini scores (GS) were calculated. Patients with the following characteristics were excluded: (1) severe congestive heart failure, malignant hypertension, severe arrhythmia, or severe lung dysfunction; (2) severe neurosis, hyperthyroidism, cervical spondylosis, hepatobiliary disease, gastric and oesophageal reflux, or chest pain caused by nonangina pectoris; (3) AMI/UAP complicated by severe primary diseases such as those of the liver, kidney, or haematopoietic system; (4) mental illness; (5) current pregnancy or lactation; (6) allergies to iodine or contrast agents or the allergic physique; and (7) various infectious diseases. Ultimately, 15 inpatients were selected and subsequently divided into the CG (control group), UAP group, and AMI group (5 patients per group). The CG was filtered according to baseline data such as the clinical CAG score (GS < 2). Then, we collected the participants' baseline data.

This study was approved by the Ethics Committee of the First Affiliated Hospital of Xinxiang Medical College (approval number: 2016039).

### 2.2. Plasma Sample Collection and CircRNA-miRNA Microarray Analysis

Five millilitres of whole blood was collected into an anticoagulant tube containing ethylenediaminetetraacetic acid (EDTA). The blood samples were stored in an ice box at 4°C and transported to the Heart Center of Xinxiang Medical University. Total RNA was extracted from 250 *μ*l of whole blood with a 750 *μ*l extraction kit (TRIpure LS Reagent, CapitalBio, Beijing, China) and was cryopreserved at −80°C. Total RNA was extracted and reverse transcribed for the synthesis of first- and second-strand cDNA. *In vitro* transcription and synthesis of cRNA were conducted, and cRNA was transcribed to generate cDNA, which was simultaneously fluorescently labelled using the Ambion WT Expression kit. A Crystal Core® CapitalBiotech Human CircRNA Array V2.0 (4 × 180 K) chip was used to analyse circRNAs. The circRNA target sequences were all from circBase (http://www.circbase.org/) and deepBase (http://rna.sysu.edu.cn/deepBase/browser.php). Human miRNA Microarray chips (8 × 60 K) (release 21.0; Agilent Technologies, Inc., Santa Clara, CA, USA) were used for the microarray analysis. The raw data were normalized by the quantile algorithm using GeneSpring Software v12.6 (Agilent Technologies, Inc.) [22, 23].

### 2.3. Statistical Analysis

Image data of the hybridized microarray (Agilent Human CircRNA Array V2.0) in tiff format were analysed by Agilent Feature Extraction (V10.7) software, and the data were extracted. Then, the circRNA array data used threshold fold change (FC) values of ≥2 and ≤−2 and a *t* test *P* value of 0.05, and miRNA array data FC ≥ 1.2 and ≤−1.2 and a *t* test *P* value of 0.05, and circRNAs and miRNAs were analysed for data summarization, normalization, and quality control by using GeneSpring GX software (Agilent). To select the differentially expressed genes, data normalization and quality control analysis were performed for each sample. CLUSTER 3.0 software was used for data analysis and graphical display. MiRanda-3.3 software was used to predict the circRNAs that may bind miRNAs and to construct a network diagram though the open-source bioinformatics software Cytoscape. In a network analysis, a degree of centrality is defined as the number of linkages one node has to another. A degree is the simplest and most important measure of gene centrality within a network for determining the relative importance. Gene ontology (GO), pathway, and disease enrichment analyses were conducted for differentially expressed circRNAs with Kyoto Encyclopedia of Genes and Genomes (KEGG) Orthology-Based Annotation System (KOBAS) software. The processing and sorting of circRNA and miRNA expression profile chip data were performed in whole or in part with the CapitalBio Technology Expression Spectrum Chip Analysis System V1.0 (computer software copyright registration number: 2014SR122558) [15, 22, 23].

Statistical analyses of baseline data were performed using SPSS 22.0, and all data are presented as the means ± standard deviations ($\bar{x}\pm s$).

## 3. Results

### 3.1. Baseline Data

Table 1 outlines the patient characteristics. Patients with AMI had lower blood pressure values and higher levels of blood glucose and myocardial enzymes than patients with UAP (*P* < 0.05). There was no significant change in blood lipid biochemistry. Patients with UAP had a calculated GS of >15 and those with AMI had a GS of >40, as evidenced by CAG. The GSs in the UAP and AMI groups were higher than those in the CG.

### 3.2. CircRNA and miRNA Expression Profiling

**Figure 1:**
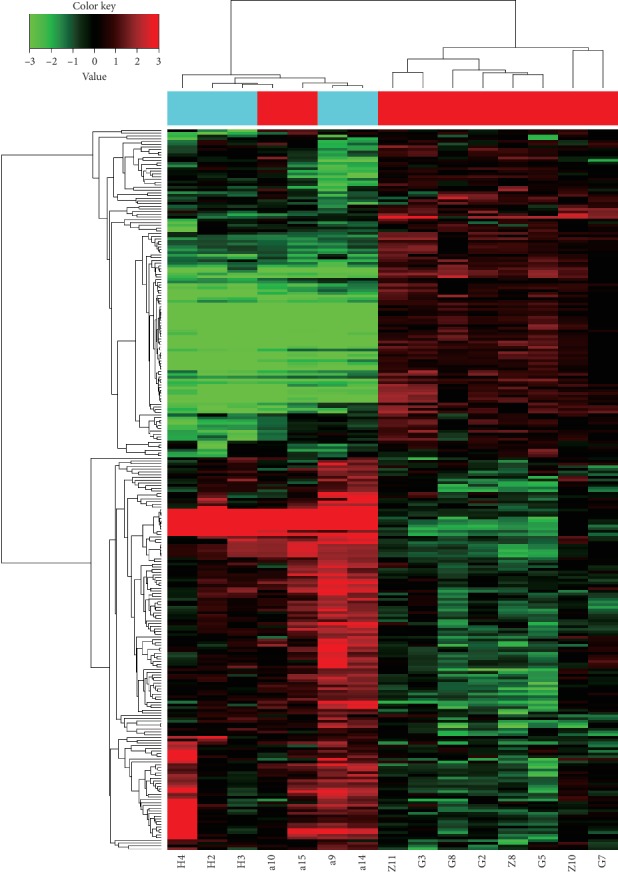
Heatmap of circRNAs with differential expression between groups. The columns represent patients, and the rows represent the degree to which a gene was expressed at different copy numbers. The colour key in the top left indicates the expression level (red indicates upregulation; green indicates downregulation). The expression of circRNAs is hierarchically clustered on the *y*-axis; the corresponding miRNAs are shown at the top. The left-most bar on the *y*-axis indicates the group assignment. The genes in 15 clusters, denoted as CG (a9, a14, H2, H3, H4), AMI (G2, G3, G5, G7, G8) and UAP (a10, a15, Z8, Z10, Z11), included 121 upregulated and 145 downregulated genes.

To identify whether circRNAs and miRNAs are differentially expressed in ACS, we extracted total RNA from the peripheral blood of 5 AMI patients, 5 UAP patients, and 5 CG with matched clinical and statistical characteristics and analysed the samples using a microarray gene chip. The detected circRNAs were differentially expressed among the groups (Figure 1). A total of 266 circRNAs (121 upregulated and 145 downregulated) were predicted in the AMI and UAP groups (*P* < 0.05, FC ≥2) (Tables 2 and 3). The data were log2-transformed and median centred by gene using the Adjust Data function in CLUSTER 3.0 software and then further analysed with hierarchical clustering with average linkage criterion (Tables 2 and 3).

Three miRNAs were differentially expressed in the AMI and UAP groups compared with those in the CG: hsa-miR-4299 was upregulated, and hsa-miR-20b-5p and hsa-miR-363-3p were downregulated (*P* < 0.05, FC ≥ 1.2) (Table 4).

### 3.3. Correlation Analysis of the circRNA-miRNA Network

CircRNAs are significant influencing factors in miRNA function and transcriptional control by acting as sponges, competing endogenous RNAs, or positive regulators of their parent coding genes. In this study, we utilized miRanda software to construct the circRNA-miRNA network, and these circRNA-miRNA pairs were screened though the open-source bioinformatics software Cytoscape. We constructed a network that matched the differentially expressed circRNAs by cross comparing the biological information among the AMI-UAP network, the AMI-CG network, the UAP-CG network, and the AMI-CG-UAP network. A large number of circRNAs that were predicted to bind to miRNAs with a combined weight score of 1 were screened as differentially expressed genes.

**Figure 2:**
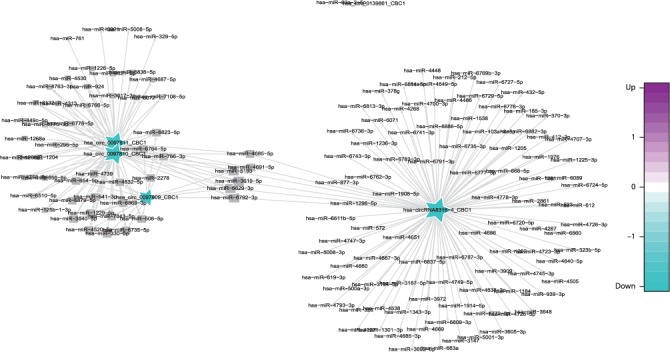
Comparison of circRNA-miRNA prediction network maps between the AMI and UAP group. Prediction of miRNAs that may be bound by circRNA and construction of a circRNA-miRNA network. According to the relationship between circRNAs and target miRNAs, the top circRNAs with the most FCs were selected to construct the circRNA-miRNA network map. The squares represent miRNAs and the pentagrams represent circRNAs, where green indicates downregulation and purple indicates upregulation.

**Figure 3:**
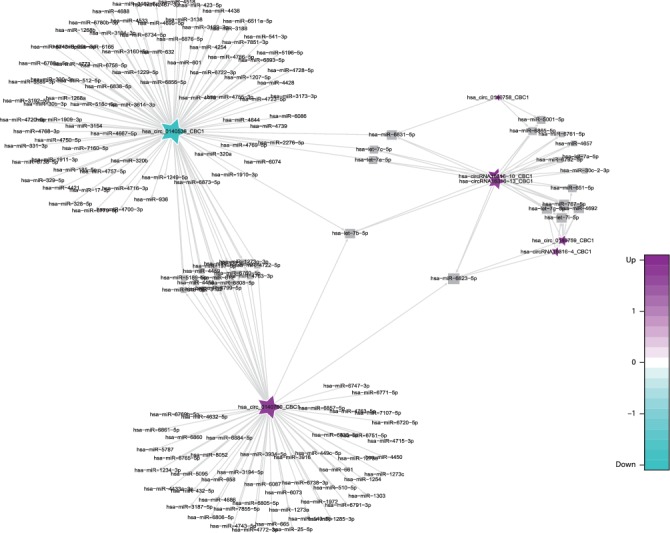
Comparison of circRNA-miRNA prediction network maps between the CG and AMI group. Prediction of miRNAs that may be bound by circRNA and construction of a circRNA-miRNA network. According to the relationship between circRNAs and target miRNAs, the top circRNAs with the most FCs were selected to construct the circRNA-miRNA network map. The squares represent miRNAs and the pentagrams represent circRNAs, where green indicates downregulation and purple indicates upregulation.

**Figure 4:**
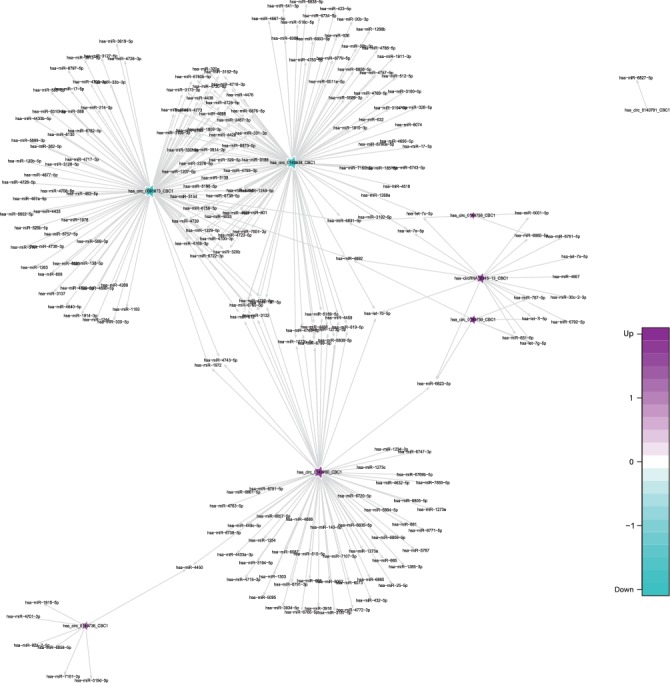
Comparison of circRNA-miRNA prediction network maps between the CG and UAP groups. Prediction of miRNAs that may be bound by circRNA and construction of a circRNA-miRNA network. According to the relationship between circRNAs and target miRNAs, the top circRNAs with the most FCs were selected to construct the circRNA-miRNA network map. The squares represent miRNAs and the pentagrams represent circRNAs, where green indicates downregulation and purple indicates upregulation.

**Figure 5:**
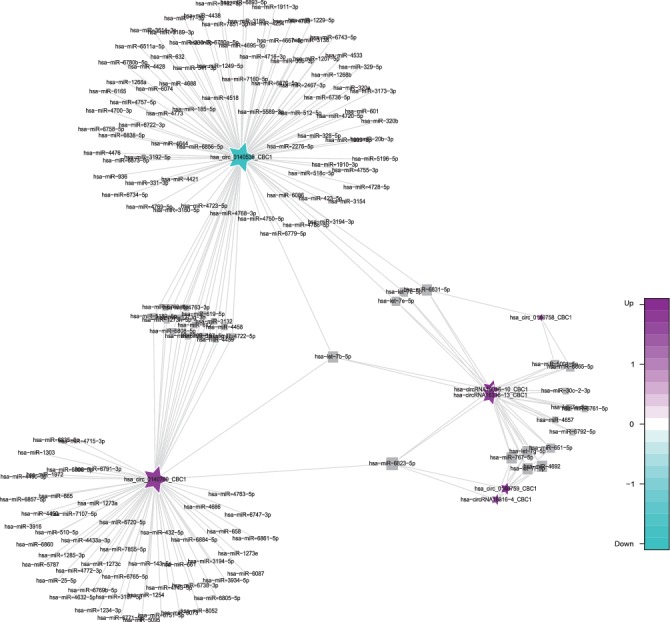
Comparison of circRNA-miRNA prediction network maps in the CG and ACS (AMI and UAP) groups. Prediction of miRNAs that may be bound by circRNA and construction of a circRNA-miRNA network. According to the relationship between circRNAs and target miRNAs, the top circRNAs with the most FCs were selected to construct the circRNA-miRNA network map. The squares represent miRNAs and the pentagrams represent circRNAs, where green indicates downregulation and purple indicates upregulation.

**Figure 6:**
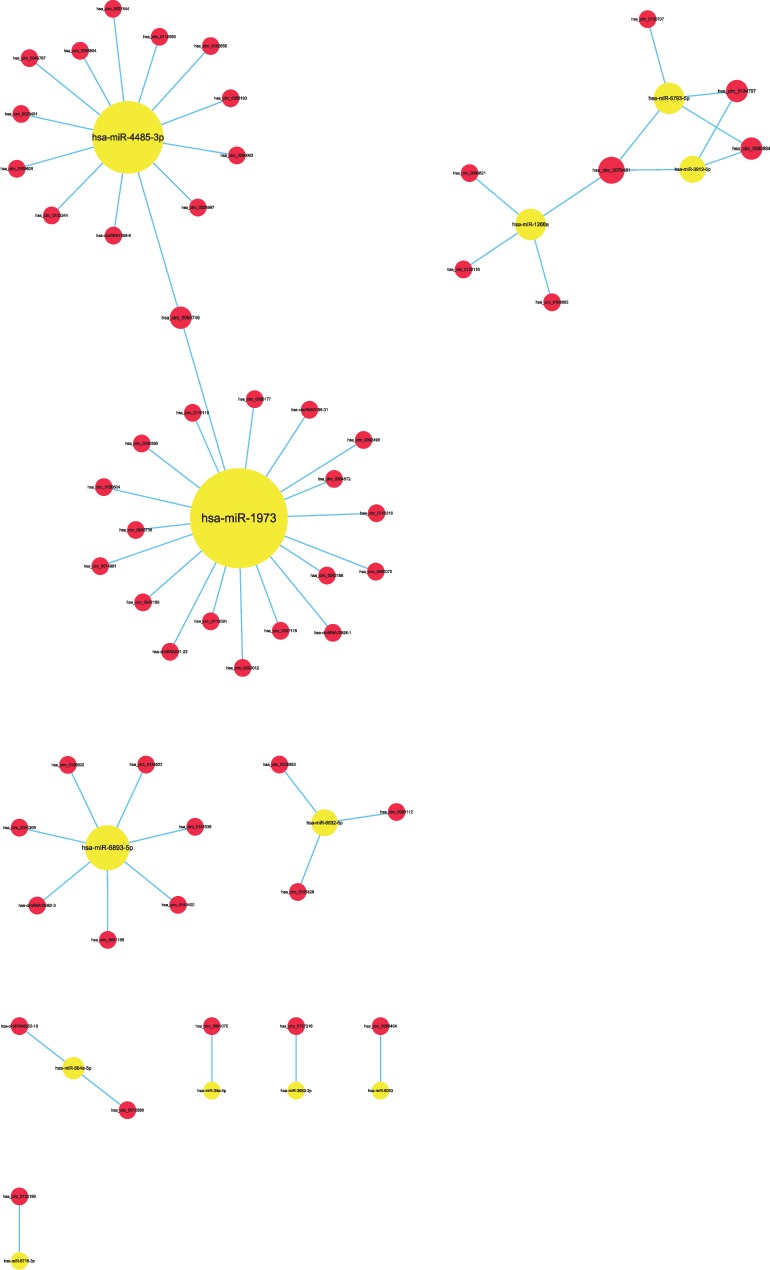
Comparison of circRNA-miRNA (red-yellow) coexpression maps between the AMI and UAP groups. These images depict the number of circRNAs that can bind to the bound target gene miRNA. The circRNAs have the same trend as miRNA changes, or the correlation is relatively close.

**Figure 7:**
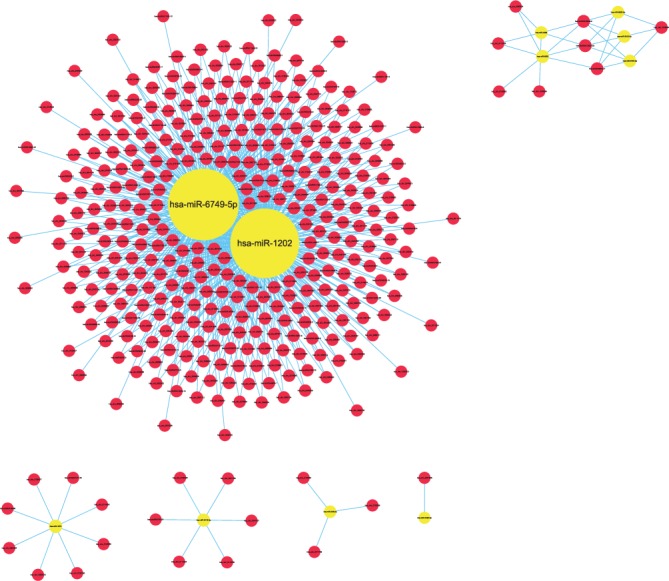
Comparison of circRNA-miRNA (red-yellow) coexpression maps between the CG and AMI groups. These images depict the number of circRNAs that can bind to the bound target gene miRNA. The circRNAs have the same trend as miRNA changes, or the correlation is relatively close.

**Figure 8:**
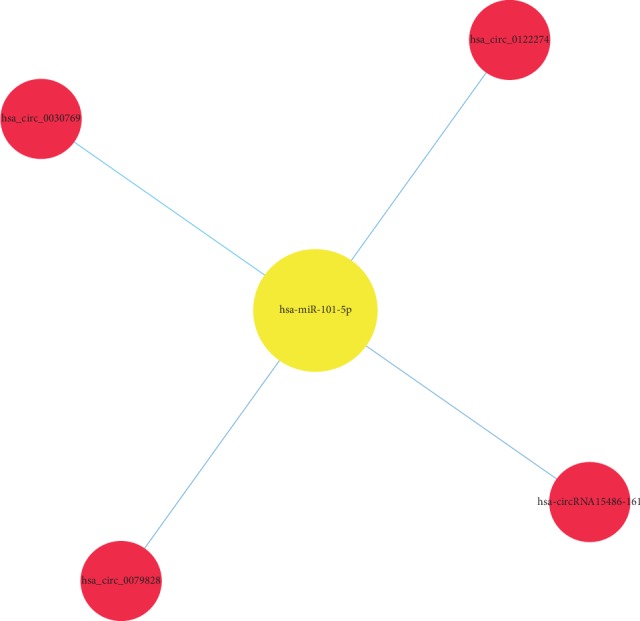
Comparison of circRNA-miRNA (red-yellow) coexpression maps between the CG and UAP groups. These images depict the number of circRNAs that can bind to the bound target gene miRNA. The circRNAs have the same trend as miRNA changes, or the correlation is relatively close.

**Figure 9:**
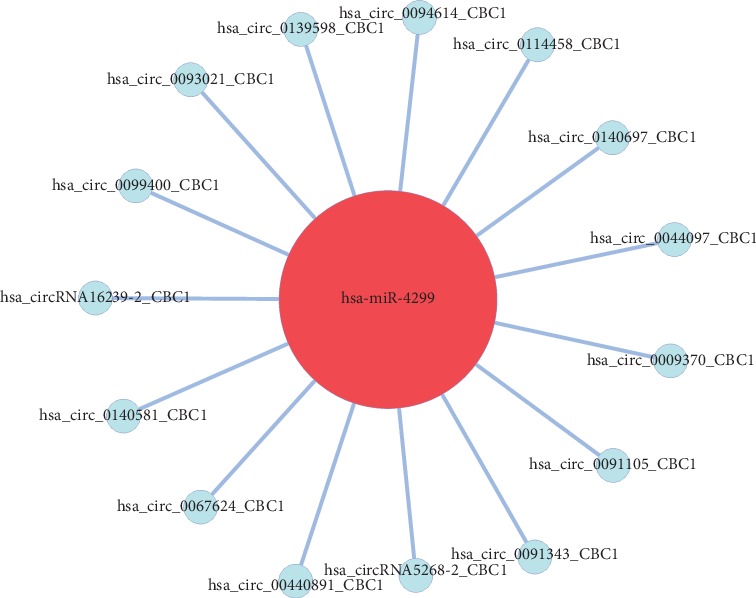
Comparison of circRNA-miRNA (blue-red) coexpression maps between the CG, AMI, and UAP groups. These images depict the number of circRNAs that can bind to the bound target gene miRNA. The circRNAs have the same trend as miRNA changes, or the correlation is relatively close.

Specifically, of the many circRNAs predicted to bind to miRNAs, not only did a single circRNA bind more than 1–100 miRNAs, but more than 1–100 circRNAs also bound a single miRNA, such as hsa_circ_8316-4, hsa-circ_0097809, hsa_circ_0097811, hsa_circ_0097810, hsa_circ_0139861, and hsa_circ_0140538, with significantly decreased expression, and hsa-circ_0140759, hsa_circ_0140758, hsa_circ_16316-13, hsa_circ_0140760, and as well as other circRNAs with significantly increased expression. Multiple miRNAs had a common locus for binding to the same circRNA, and vice versa. These results suggested that circRNAs exhibited coexpression correlations with miRNAs and that circRNAs could act as source genes to interact with miRNAs to regulate the occurrence and development of ACS (Figures 2–5). All differentially expressed circRNAs are presented in Table 5. Simultaneously, miRNAs could act as target genes to interact with copious circRNAs (Table 6, Figures 6–9). However, how these novel genes participate in the process of disease development is still unclear. Further cells culture or animal experiments are needed to verify these findings.

### 3.4. CircRNA Enrichment Analyses

**Figure 10:**
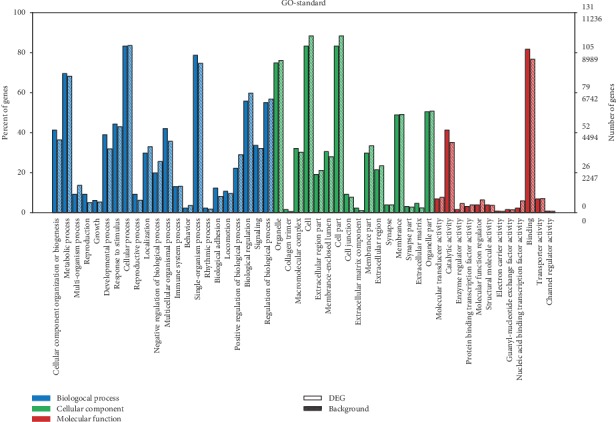
Functional annotation of differentially expressed genes (DEGs). Itshows the enrichment of DEGs compared with the background genes.

**Figure 11:**
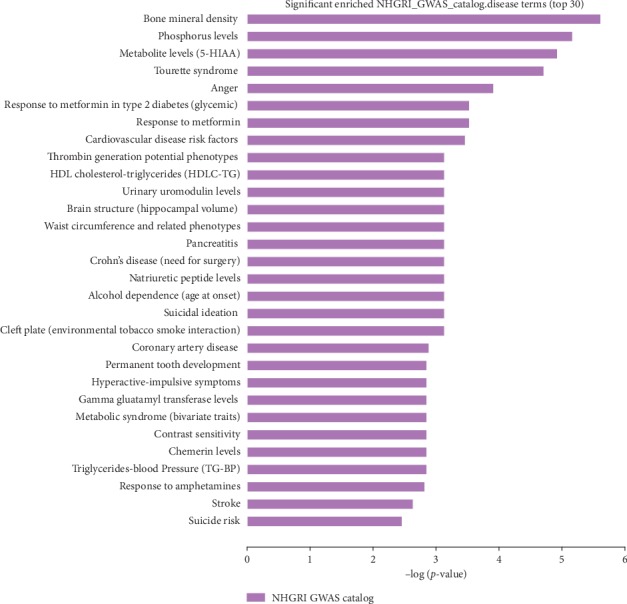
Functional annotation of differentially expressed genes (DEGs). Disease ontology analysis was used to select the top 30 terms that were significantly enriched, and the *P* values were then used to sort the top plots.

**Figure 12:**
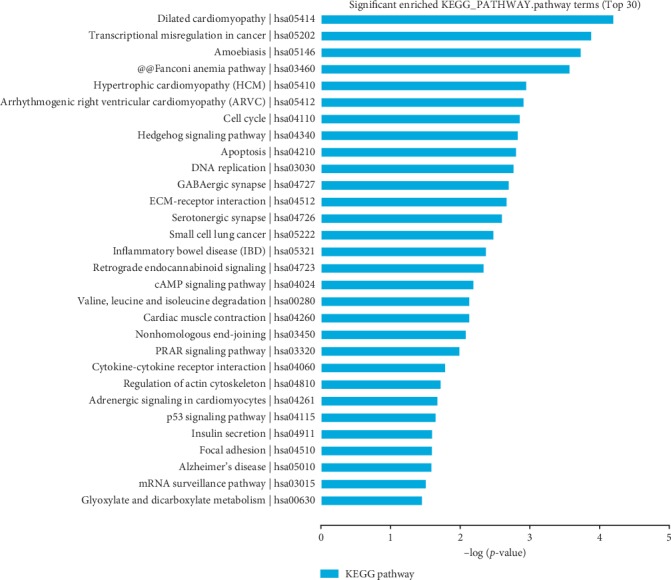
Functional annotation of differentially expressed genes (DEGs). The KEGG pathway analysis was used to select the top 30 terms that were significantly enriched, and the *P* values were then used to sort the top plots.

To better understand the functions of the genes associated with the differentially expressed circRNAs, GO (Figure 10), disease (Figure 11), and pathway (Figure 12) enrichment analyses were performed with KOBAS software. Pathway and disease terms were selected from the first 30 significantly enriched terms. GO analysis was used to select the first 30 significantly enriched biological process (BP), molecular function (MF), and cellular component (CC) terms, and a histogram was drawn according to the *P* values, which directly reflected the significantly enriched terms.

### 3.5. GO Enrichment Analysis

We carried out KEGG pathway mapping based on the encyclopaedia's orthology terms to assess related pathways correlating with differentially expressed circRNAs from the AMI and UAP groups compared with those from the CG. A total of 53 MF terms, 241 BP terms, and 35 CC terms were significantly enriched (*P* < 0.05). These three major categories define and describe various aspects of a gene's function. We selected the first 30 significantly enriched terms from the three categories and plotted a histogram according to the *P* values, which directly reflect the significantly enriched terms (*P* < 0.05; Figure 10). The top-ranking GO terms involved in ACS included the metabolic process (GO:0008152), cellular process (GO:0009987), single organism process (GO:0044699), organelle (GO:0043226), cell (GO:0005623), cell part (GO:0044464), and binding (GO:0005488).

### 3.6. Disease Enrichment Analysis

Disease enrichment analysis was performed using the NHGRI GWAS Catalog. A total of 19 terms were significantly enriched (*P* < 0.05). The diseases predicted to be associated with ACS included cardiovascular disease risk factors and high-density lipoprotein cholesterol (HDL-C).

The differentially expressed circRNAs were found to be involved in terms related to cardiovascular diseases, such as bone mineral density (hsa123803), phosphorus levels (hsa221496), metabolite levels (5−HIAA) (hsa4023), tourette syndrome (hsa5251), and anger (hsa56776) (*P* < 0.05; Figure 11).

### 3.7. Pathway Enrichment Analysis

In the KEGG pathway database, 266 circRNAs with different expression levels were enriched, and the first 30 significantly enriched terms were selected (*P* < 0.05). We found pathways that may be involved in ACS: dilated cardiomyopathy (hsa05141), transcriptional misregulation in cancer (hsa05202), amoebiasis (hsa05146), Fanconi anaemia pathway (hsa03460), hypertrophic cardiomyopathy (hsa05410), and arrhythmogenic right-ventricular cardiomyopathy (hsa05412) (*P* < 0.05; Figure 12).

## 4. Discussion

Accumulating evidence suggests that ncRNA participates in diseases such as cardiovascular diseases, diabetes mellitus, and hypertension. Recent progress in the ncRNA research field has uncovered central aspects for the regulation and functions of biogenesis and biology in the pathophysiology of the cardiovascular system [11, 12]. More importantly, the circRNA-miRNA-mRNA regulatory network plays an important role in the occurrence and development of cardiovascular disease [11].

The research results reported in this paper offer many important findings and imply that circRNA and miRNA are differentially expressed between the ACS groups and the CG. Differential expression was detected for a total of 266 circRNAs, of which 121 were upregulated and 145 were downregulated in the ACS groups (*P* < 0.05, FC ≥ 2), and the circRNAs were found to be related to UTY, KDM5D, USP9Y, MRPL39, ABCA5, and CCT8 and as well as other genes (Tables 2 and 3). In previous studies, a total of 1670 circRNAs were identified in the AMI group (859 upregulated and 811downregulated) and a total of 110 circRNAs were identified in the CHD group (73 upregulated and 73 downregulated) (*P* < 0.05, FC ≥ 2.0). Furthermore, hsa_circ_16316-13 was found to be significantly increased in CHD patients [18, 24]; similarly, hsa _circ_16316-13 was found to be upregulated in this paper.

To date, many outcomes have confirmed the view that circRNAs can be used as sponges for miRNA to regulate the gene expression. Recent evidence points to a pivotal role for circRNAs in the regulation of miRNA function as miRNA sponges; in addition, they may play a significant role in pathophysiology of cardiovascular diseases [25]. Strikingly, genetic network bioinformatics analysis for ACS revealed not only that a single circRNA could bind to more than 1–100 miRNAs but also more than 1–100 circRNAs bound to a single miRNA (Figures 2–9). The results provide evidence to show that circRNA is bound with miRNA in the occurrence and development of ACS. Another study showed that circRNA_101237 acts as a sponge for let-7a-5p, regulating cardiomyocyte death and autophagy; additionally, the circRNA-101237/let-7a-5p/IGF2BP3 (insulin-like growth factor 2 mRNA-binding protein 3) axis serves as a regulator of cardiomyocyte death [26]. CircNCX1 was increased in response to reactive oxygen species and promotes cardiomyocyte apoptosis by competitive binding to miR-133a-3p, suppressing the activity of CDIP1 (a proapoptotic gene cell death-inducing protein) by acting as an endogenous miR-133a-3p sponge [27]. Thus, circRNA-miRNA axes are involved in a series of disease pathways such as myocardial infarction, myocardial hypertrophy, cardiac regeneration, cardiac fibroblasts, and heart failure [2, 25, 28]. Moreover, targeted localization of circRNA-miRNA-mRNA may be a potential target for cardiovascular disease treatment [16].

The results of the KEGG pathway enrichment analysis revealed that differentially expressed genes are involved in the ACS signalling pathway, such as dilated cardiomyopathy, hypertrophic cardiomyopathy, and arrhythmogenic right-ventricular cardiomyopathy (Figure 12). The dilated cardiomyopathy (DCM) pathway involves proteins such as Desmin, DMD, Titin, Tnt, ACTA1, TPM, Laminac, SGCD, TNF-*α*, IGF-1, TGF-*β*, and Ang-II. Additionally, more valuable in current clinical practice, cTnT is the preferred biochemical marker for myocardial cell necrosis, and alleviated cTnT levels are detected only 3–6 hours after the onset of ischaemic symptoms. Currently, a positive troponin result is associated with clinically important increases in mortality, regardless of age, even if the level is only slightly above normal [29]. In addition, the pathway is related to arrhythmogenic right-ventricular cardiomyopathy in ACS (Figure 12).

Among the results of the enrichment analysis of 155 diseases, 19 diseases were predicted to be associated with ACS (Figure 11), such as cardiovascular disease risk factors and HDL-C. Risk factors for ACS are known to include hypertension, hyperlipidaemia, triglycerides, smoking, alcoholism, diabetes, lack of exercise, anger, overweight, and genetic factors. In recent years, many basic science and clinical studies have reported that glycaemic variability [30], impaired spontaneous/endogenous fibrinolytic status [31], low-density lipoprotein (LDL), nonadherence [32], socioeconomic and psychosocial factors, grip strength, household environment, ambient pollution, and sodium intake [33] are highly significant risk factors for cardiovascular disease.

Encouragingly, some of these differentially expressed genes have been validated in cardiovascular diseases. Clinical research conducted by Vilade et al. revealed that hsa_circ_0001445 exists stably in plasma and can serve as a new biomarker for coronary artery disease, as it was associated with a higher extent of coronary atherosclerosis [34]. Recent reports have also suggested that circRNA is another type of large noncoding RNA with translation potential [35–37]. Sebastiaan van Heesch et al. focused on protein translation in the heart for the first time using a method combining ribosomal imprinting and provided information on protein translation regulation during DCM [38]. Experiments on mice carried out by Garikipati VNS et al. revealed that overexpression of circFndc3b contributed to reducing myocardial and endothelial cell apoptosis and improving myocardial function. Furthermore, circFndc3b interacted with the RNA-binding protein FUS to positively regulate the expression of VEGF-A, thereby improving the function and reconstruction of the myocardium after infarction [39].

In conclusion, circRNAs are involved in the occurrence and development of ACS through multiple points of network correlation for miRNA regulation. We speculate that circRNAs may serve as a potential therapeutic avenue for a pathophysiological mechanism of ACS and may even become diagnostic and therapeutic biomarkers for ACS. In the future, we will perform *in vitro* and *in vivo* tests to further validate the involvement of circRNAs in the atherosclerotic process of ACS.

## Figures and Tables

**Table 1:** Patients characteristics ($\;\bar{x}\pm s$ group *=* 5).

	CG	UAP	AMI
Age (years)	55 ± 11	60 ± 11	54 ± 8
HR (min)	66 ± 9	71 ± 13	81 ± 10
SBP (mmHg)	149 ± 10	146 ± 11	130 ± 15
DBP (mmHg)	83 ± 12	79 ± 16	75 ± 6
Glu (3.9–6.1 mmol/L)	5.25 ± 7.82	5.34 ± 0.62	6.46 ± 1.92
CHO (0–5.2 mmol/L)	3.69 ± 0.88	3.99 ± 1.05	3.81 ± 0.37
TG (0.7–1.7 mmol/L)	1.26 ± 0.65	1.21 ± 0.41	1.27 ± 0.45
APOA1 (1–1.76 g/L)	1.13 ± 0.20	1.00 ± 0.10	0.89 ± 0.12
APOB (0.6–1.14 g/L)	0.72 ± 0.20	0.79 ± 1.84	0.71 ± 0.15
HDL (0.8–1.55 mmol/L)	1.11 ± 0.18	0.92 ± 0.20	0.95 ± 0.12
LDL(1.64–3.62 mmol/L)	2.25 ± 0.66	2.54 ± 0.92	2.52 ± 0.44
AST (15–50 U/L)	21.60 ± 4.98	25.60 ± 4.22	96.80 ± 65.06
LDH (313–618 U/L)	149.20 ± 17.20	162.60 ± 29.59	449.00 ± 265.70
CK (55–170 U/L)	48.00 ± 13.21	70.80 ± 35.09	817.60 ± 535.16
CK-MB (0–25 U/L)	15.80 ± 7.22	16.80 ± 4.23	74.40 ± 48.55
Gensini score (%)	1.00 ± 1.41	65.40 ± 49.09	51.90 ± 10.37

APOA1 = apolipoprotein A1; APOB = apolipoprotein B; CHO = total cholesterol; HDL = high-density lipoprotein; LDL = low-density lipoprotein; AST = aspartate transaminase; LDH = lactate dehydrogenase; CK = creatine kinase; CK-MB = creatine kinase isoenzyme MB.

**Table 2:** Expression profiles of 121 circRNAs that were upregulated (*P* < 0.05, FC ≥ 2).

No.	ProbeName	*P*	FC (abs)	geneSymbol	circStart	circEnd	Strand	miRNA number	miRNA number more than 1
1	hsa-circ16316-10	0.00183	156.1532	UTY	15447442	15481229	−	100	17
2	hsa_circ0140759	0.00078	145.55	UTY	15466882	15481229	−	95	6
3	hsa_circ0140760	0.00139	93.25378	UTY	15467172	15471765	−	100	66
4	hsa-circ16316-13	0.00141	88.45529	UTY	15447442	15478273	−	100	17
5	hsa_circ0140758	0.00147	80.8945	UTY	15447442	15448215	−	100	3
6	hsa-circ16316-4	0.00199	61.61567	UTY	15466882	15472408	−	85	5
7	hsa-circ16316-11	0.00116	44.99736	UTY	15471646	15471866	−	11	—
8	hsa_circ0140781	0.00129	26.7885	KDM5D	21901413	21903743	−	49	1
9	hsa_circ0140736	0.00149	24.01664	USP9Y	14821320	14885859	+	100	7
10	hsa_circ0003368	0.00689	22.6279	UTY	15478146	15481229	−	9	—
11	hsa-circ16316-9	0.00064	21.13425	UTY	15435434	15438230	−	43	4
12	hsa_circ0009024	0.00047	17.24446	—	21749095	21749393	+	25	—
13	hsa_circ0140746	0.00106	16.81741	USP9Y	14870435	14885859	+	42	—
14	hsa-circ16316-2	0.00316	16.45998	UTY	15435434	15481229	−	100	24
15	hsa-circ16316-12	0.00040	15.87258	UTY	15435434	15448215	−	100	7
16	hsa_circ0140783	0.00160	15.52771	—	22669237	22683186	−	100	100
17	hsa-circ16316-1	0.01056	14.98803	UTY	15478146	15508852	−	12	—
18	hsa_circ0007907	0.00053	13.54894	ZFY	2829114	2829687	+	25	—
19	hsa_circ0140780	0.00062	12.87783	KDM5D	21901413	21901548	−	12	—
20	hsa-circ16316-7	0.00914	12.75820	UTY	15522872	15526673	−	15	—
……	……		……						
121	hsa_circ0076034	0.00770	2.00799	LEMD2	33744730	33756906	−	100	100

ProbeName = probe address; FC = fold change; geneSymbol = abbreviated gene name; circStart = gene initiation site; circEnd = gene termination position; strand = circRNA in chain; miRNA number = number of miRNAs that the circRNA can bind to (sorted by the number of binding sites—if greater than 100, only the top 100 binding sites are selected); miRNA number more than 1 = the circRNA can bind to 2 or more miRNAs.

**Table 3:** Expression profiles of 145 circRNAs that were downregulated (*P* < 0.05, FC ≥ 2).

No.	ProbeName	*P*	FC (abs)	geneSymbol	circStrat	circEnd	Strand	miRNA number	miRNA number more than 1
1	hsa_circ0140537	0.001857	66.81375	—	73044457	73044598	−	6	—
2	hsa_cir_0091074	0.001099	62.43283	—	73048902	73051109	−	59	—
3	hsa_circ0140538	0.001115	53.70956	—	73045949	73051109	−	100	100
4	hsa_circ0091073	0.001317	38.93487	—	73040494	73051109	−	100	100
5	hsa_circ0140539	0.001931	27.13039	—	73045949	73057338	−	100	100
6	hsa-circ16166-3	0.001541	23.23273	—	73050900	73053209	−	39	—
7	hsa-circ16166-1	0.001702	20.40522	—	73050900	73057338	−	50	1
8	hsa_circ0140540	0.001844	15.58513	—	73045949	73061308	−	100	100
9	hsa_circ0140536	0.001075	13.07049	—	73044087	73044570	−	16	—
10	hsa_circ0140541	0.00105	6.618035	—	73046801	73046954	−	11	—
11	hsa-circ13156-4	0.029169	5.155549	MRPL39	26966204	26976247	−	39	—
12	hsa_circ0107597	0.005089	4.704399	ABCA5	67270099	67305564	−	100	6
13	hsa_circ0061370	0.028818	4.348159	CCT8	30428647	30434877	−	73	—
14	hsa_circ0058143	0.027599	4.206255	FN1	216279383	216286966	−	92	3
15	hsa_circ0003086	0.024972	4.089472	—	64630738	65139334	+	100	100
16	hsa_circ0140697	0.038202	3.944226	KLHL4	86919763	86924916	+	100	36
17	hsa_circ0052193	0.018474	3.741495	PTPRH	55692614	55699536	−	100	87
18	hsa_circ0140547	0.01636	3.64521	—	73071865	73072197	+	42	5
19	hsa_circ0140553	0.019321	3.624559	—	73071957	73072197	+	38	4
20	hsa_circ0140549	0.018403	3.623656	—	73071909	73072197	+	41	5
……									
145	hsa_circ_0046285	0.046716	2.004205	PYCR1	79890268	79894968	−	100	100

**Table 4:** MiRNAs expression profiling (*P* < 0.05, FC ≥ 1.2).

No.	ProbeName	*P*	FC (abs)	Regulation
1	Hsa-miR-4299	0.016	9.07	Up
2	Hsa-miR-20b-5p	0.033	1.29	Down
3	Hsa-miR-363-3p	0.046	1.21	Down

ProbeName = probe address; FC = fold change; There shows miRNAs that were differentially expressed between the groups.

**Table 5:** Differential expression of circRNAs in circRNA-miRNA network^*∗*^.

No.	Gene expression	AMI vs UAP	CG vs AMI	CG vs UAP	CG, AMI, and UAP
1	Upregulated		Hsa_circ_0140758	hsa_circ_0140758	hsa_circ_0140758
2			Hsa-circ_0140759	hsa_circ_0140759	hsa-circ_0140759
3			Hsa_circ_0140760	hsa_circ_0140760	hsa_circ_0140760
4			Hsa_circ_16316-13	hsa-circ_16316-13	hsa_circ_16316-13
5			Hsa_circ_16316-10	hsa_circ_0140736	hsa_circ_16316-10
6			Hsa-circ_16316-4	hsa_circ_0140781	hsa-circ_16316-4

1	Downregulated	hsa_circ_8316-4	Hsa_circ_0140538	hsa_circ_0140538	hsa_circ_0140538
2		hsa-circ_0097809		hsa_circ_0091073	
3		hsa_circ_0097811			
4		hsa_circ_0097810			
		hsa_circ_0139861			

^*∗*^ *P* < 0.05, up FC ≥ 2, down FC ≥ −2.

**Table 6:** Analysis of the number of source genes (circRNA) and the number of target gene miRNAs.

	AMI and UAP		CG and UAP		AMI and CG		CG, AMI, and UAP	
No.	SGS (No.)	TGS	SGS (No.)	TGS	SGS (No.)	TGS	SGS (No.)	TGS
1	10	hsa-miR-6832-5p	4	hsa-miR-101-5p	5	hsa-miR-4299	15	hsa-miR-4299
2	19	hsa-miR-1973			4	hsa-miR-6832-5p		
3	13	hsa-miR-4485-3p			8	hsa-miR-1973		
4	2	hsa-miR-664a-5p			1	hsa-miR-4485-3p		
5	3	hsa-miR-3912-5p			4	hsa-miR-3912-5p		
6	1	hsa-miR-8063			6	hsa-miR-8063		
7	1	hsa-miR-3663-3p			4	hsa-miR-6793-5p		
8	4	hsa-miR-1268a			331	hsa-miR-6749-5p		
9	4	hsa-miR-6793-5p			3	hsa-miR-328-5p		
10	1	hsa-miR-34a-5p			6	hsa-miR-6716-3p		
11	1	hsa-miR-6716-3p			320	hsa-miR-1202		

geneSymbol is the gene abbreviation. SGS: source geneSymbol; TGS: target geneSymbol.

## Data Availability

The datasets used and/or analysed during the current study are available from the corresponding author upon reasonable request.

## Additional Points

*Highlights*. (a) CircRNA and miRNA may be closely related to the pathogenesis of acute coronary syndrome. (b) Based on gene chip analysis, 266 circRNAs and 3 miRNAs were differentially expressed in acute coronary syndrome. (c) A single circRNA can bind to more than 1–100 miRNAs, and vice versa. (d) The metabolism pathways, signalling pathways, and involved diseases affected by these circRNAs were predicted by enrichment analysis.

## References

[1] Li X., Yang L., Chen L.-L. (2018). The biogenesis, functions, and challenges of circular RNAs. *Molecular Cell*.

[2] Aufiero S., Reckman Y. J., Pinto Y. M., Creemers E. E. (2019). Circular RNAs open a new chapter in cardiovascular biology. *Nature Reviews Cardiology*.

[3] Kristensen L. S., Andersen M. S., Stagsted L. V. W., Ebbesen K. K., Hansen T. B., Kjems J. (2019). The biogenesis, biology and characterization of circular RNAs. *Nature Reviews Genetics*.

[4] Jeck W. R., Sorrentino J. A., Wang K. (2013). Circular RNAs are abundant, conserved, and associated with ALU repeats. *RNA*.

[5] Zhang Y., Zhang X.-O., Chen T. (2013). Circular intronic long noncoding RNAs. *Molecular Cell*.

[6] Li Z., Huang C., Bao C. (2015). Exon-intron circular RNAs regulate transcription in the nucleus. *Nature Structural & Molecular Biology*.

[7] Braicu C., Zimta A.-A., Gulei D., Olariu A., Berindan-Neagoe I. (2019). Comprehensive analysis of circular RNAs in pathological states: biogenesis, cellular regulation, and therapeutic relevance. *Cellular and Molecular Life Sciences*.

[8] Lei M., Zheng G., Ning Q., Zheng J., Dong D. (2020). Translation and functional roles of circular RNAs in human cancer. *Molecular Cancer*.

[9] Mehta S. L., Dempsey R. J., Vemuganti R. (2020). Role of circular RNAs in brain development and CNS diseases. *Progress in Neurobiology*.

[10] Zhou M.-y., Yang J.-M., Xiong X.-d. (2018). The emerging landscape of circular RNA in cardiovascular diseases. *Journal of Molecular and Cellular Cardiology*.

[11] Lu D., Thum T. (2019). RNA-based diagnostic and therapeutic strategies for cardiovascular disease. *Nature Reviews Cardiology*.

[12] Beermann J., Piccoli M.-T., Viereck J., Thum T. (2016). Non-coding RNAs in development and disease: background, mechanisms, and therapeutic approaches. *Physiological Reviews*.

[13] Salzman J. (2016). Circular RNA expression: its potential regulation and function. *Trends in Genetics*.

[14] Chen Y., Li C., Tan C., Liu X. (2016). Circular RNAs: a new frontier in the study of human diseases. *Journal of Medical Genetics*.

[15] Memczak S., Jens M., Elefsinioti A. (2013). Circular RNAs are a large class of animal RNAs with regulatory potency. *Nature*.

[16] Zhao G. (2018). Significance of non-coding circular RNAs and micro RNAs in the pathogenesis of cardiovascular diseases. *Journal of Medical Genetics*.

[17] Jakob P., Kacprowski T., Briand-Schumacher S. (2017). Profiling and validation of circulating microRNAs for cardiovascular events in patients presenting with ST-segment elevation myocardial infarction. *European Heart Journal*.

[18] Lin F., Zhao G., Chen Z. (2019). circRNAmiRNA association for coronary heart disease. *Molecular Medicine Reports*.

[19] Thygesen K., Alpert J. S., Jaffe A. S. (2019). Fourth universal definition of myocardial infarction (2018). *European Heart Journal*.

[20] Writing Committee M., Jneid H., Anderson J. L. (2012). ACCF/AHA focused update of the guideline for the management of patients with unstable angina/Non-ST-elevation myocardial infarction (updating the 2007 guideline and replacing the 2011 focused update): a report of the American College of Cardiology Foundation/American Heart Association task force on practice guidelines. *Circulation*.

[21] Fihn S. D., Blankenship J. C., Alexander K. P. (2014). 2014 ACC/AHA/AATS/PCNA/SCAI/STS focused update of the guideline for the diagnosis and management of patients with stable ischemic heart disease. *Circulation*.

[22] You X., Vlatkovic I., Babic A. (2015). Neural circular RNAs are derived from synaptic genes and regulated by development and plasticity. *Nature Neuroscience*.

[23] Patterson T. A., Lobenhofer E. K., Fulmer-Smentek S. B. (2006). Performance comparison of one-color and two-color platforms within the MicroArray Quality Control (MAQC) Project. *Nature Biotechnology*.

[24] Lin F Z. G., Chen Z. G., Wang X. H (2018). Network correlation of circRNA-miRNA and the possible regulatory mechanism in acute myocardial infarction. *Zhonghua Yi Xue Za Zhi*.

[25] Lin F., Chen H.-W., Zhao G.-A. (2020). Advances in research on the circRNA-miRNA-mRNA network in coronary heart disease treated with traditional Chinese medicine. *Evidence-based Complementary and Alternative Medicine*.

[26] Gan J., Yuan J., Liu Y. (2020). Circular RNA_101237 mediates anoxia/reoxygenation injury by targeting let7a5p/IGF2BP3 in cardiomyocytes. *International Journal of Molecular Medicine*.

[27] Li M., Ding W., Tariq M. A. (2018). A circular transcript of ncx1 gene mediates ischemic myocardial injury by targeting miR-133a-3p. *Theranostics*.

[28] Gomes C. P. d. C., Schroen B., Kuster G. M. (2020). Regulatory RNAs in heart failure. *Circulation*.

[29] Kaura A., Panoulas V., Glampson B. (2019). Association of troponin level and age with mortality in 250 000 patients: cohort study across five UK acute care centres. *BMJ*.

[30] Gerbaud E., Darier R., Montaudon M. (2019). Glycemic variability is a powerful independent predictive factor of midterm major adverse cardiac events in patients with diabetes with acute coronary syndrome. *Diabetes Care*.

[31] Gorog D. A., Lip G. Y. H. (2019). Impaired spontaneous/endogenous fibrinolytic status as new cardiovascular risk factor?. *Journal of the American College of Cardiology*.

[32] Kones R., Rumana U., Morales-Salinas A. (2019). Confronting the most challenging risk factor: non-adherence. *The Lancet*.

[33] Yusuf S., Joseph P., Rangarajan S. (2019). Modifiable risk factors, cardiovascular disease, and mortality in 155 722 individuals from 21 high-income, middle-income, and low-income countries (PURE): a prospective cohort study. *The Lancet*.

[34] Vilades D., Martínez‐Camblor P., Ferrero‐Gregori A. (2020). Plasma circular RNA hsa_circ_0001445 and coronary artery disease: performance as a biomarker. *The FASEB Journal*.

[35] Legnini I., Di Timoteo G., Rossi F. (2017). Circ-ZNF609 is a circular RNA that can Be translated and functions in myogenesis. *Molecular Cell*.

[36] Yang Y., Fan X., Mao M. (2017). Extensive translation of circular RNAs driven by N6-methyladenosine. *Cell Research*.

[37] Abdelmohsen K., Panda A. C., Munk R. (2017). Identification of HuR target circular RNAs uncovers suppression of PABPN1 translation by CircPABPN1. *RNA Biology*.

[38] Van Heesch S., Witte F., Schneider-Lunitz V. (2019). The translational landscape of the human heart. *Cell*.

[39] Garikipati V. N. S., Verma S. K., Cheng Z. (2019). Circular RNA CircFndc3b modulates cardiac repair after myocardial infarction via FUS/VEGF-A axis. *Nature Communications*.

